# Replacing alfalfa hay with amaranth hay: effects on production performance, rumen fermentation, nutrient digestibility and antioxidant ability in dairy cow

**DOI:** 10.5713/ab.23.0232

**Published:** 2023-11-01

**Authors:** Jian Ma, Xue Fan, Guoqing Sun, Fuquan Yin, Guangxian Zhou, Zhihui Zhao, Shangquan Gan

**Affiliations:** 1 College of Coastal Agricultural Sciences, Guangdong Ocean University, Zhanjiang 524088, China; 2 College of Life Sciences, Northwest Normal University, Lanzhou 730070, China; 3 Modern Animal Husbandry (Hefei) Co. Ltd., Hefei 230000, China

**Keywords:** Amaranth Hay, Dairy Cow, Nutrient Digestibility, Production Performance, Rumen Fermentation

## Abstract

**Objective:**

The aim of this research was to explore the effects of dietary substitution of alfalfa hay by amaranth hay on production performance, rumen fermentation, nutrient digestibility, serum biochemical parameters and antioxidant ability in dairy cows.

**Methods:**

A total of 45 healthy Holstein cows with same parity and similar milk yield and body weight were randomly divided into 3 groups: control diet without amaranth hay (CON) or 50% and 100% alfalfa hay replaced by an equal amount of amaranth hay (dry matter basis, AH1 and AH2, respectively). All the cows were fed regularly 3 times a day at 06:30, 14:30, and 22:30 and had free access to water. The experiment lasted for 60 d.

**Results:**

The dry matter intake of CON and AH1 groups was higher (p<0.05) than that of AH2 group. Compared with AH1 group, the milk yield of AH2 group was reduced (p<0.05). Moreover, dietary substitution of alfalfa hay by amaranth hay increased (p<0.05) milk fat, ammonia nitrogen and acetate concentrations. However, the crude protein digestibility of AH2 group was lower (p<0.05) than that of CON group, while an opposite tendency of serum urea nitrogen was found between two groups. The neutral detergent fiber digestibility of AH1 group was increased (p<0.05) when compared to AH2 group. Amaranth hay treatment increased (p<0.05) the serum concentration of glutathione peroxidase in dairy cows. Compared with CON group, the malonaldehyde activity of AH1 group was decreased (p<0.05).

**Conclusion:**

Dietary replacing alfalfa hay with amaranth hay (50% ratio) in dairy cows did not affect production performance but improved their antioxidant ability.

## INTRODUCTION

The roughage is a key ingredient in the diet of dairy cows because of the special physiological characteristics of ruminant gastrointestinal tract. A sufficient supply of roughage of high quality is critical for maintaining ruminal health [1]. Alfalfa hay is a commonly used roughage in the diet of dairy cows. However, alfalfa has a high requirement for water. Due to the limited land resources, water scarcity and poor soils, the yield of some fodder crops (e.g. alfalfa) is poor in some parts of the world. Moreover, the price of imported alfalfa hay is higher. The shortage of roughage has become an important factor restraining the development of dairy industry in some regions. Therefore, finding roughage that does not negatively influence the production performance of dairy cows is of great importance in dairy farming. In recent years, as alternative roughage, the utilization of non-conventional feed resources with high crude protein (CP) content, nutrients digestibility and yield has attracted increasing attention.

Some crops which have the ability to adapt to lack of water, high temperature and poor soil can be utilized as roughage resources for ruminants under harsh conditions [2,3]. The amaranth (*Amaranthus hypochondriacus*), a C_4_ dicotyledonous crop, can grow in the regions with these harsh conditions. Forage amaranth has the characteristics of high yield performance and nutritional value. According to our previous investigation, the yield of amaranth harvested at heading stage reached up to 130 t/hm^2^ (fresh weight) and 20 t/hm^2^ (air-dry matter), respectively [4]. Compared with other crops (e.g. corn), the amaranth has lower contents of lignin, oxalic acid and nitrate [5]. In addition, the CP content of whole amaranth plant is approximately 14% when it is harvested at the budding stage [4]. Because of these advantages mentioned, the amaranth can be used as a roughage in ruminant production. Previous studies in dairy cows [6] and fattening lambs [7] have reported that the partial replacement of corn silage with amaranth silage in the diet did not have negative influence on health and production performance of animals and could decrease the feeding cost. However, the utilization of amaranth hay in ruminant production has not fully investigated.

Oxidative stress damage has many adverse consequences on productivity and immunity and affects growth development and health of dairy cows. Antioxidant substances, such as vitamin C and carotene, in amaranth can destroy free radicals and enhance the activity of antioxidant enzymes. On the other hand, the polyphenols (e.g. rutin) can inhibit the oxidation of high density lipoprotein cholesterol, which further enhance the antioxidant capacity of amaranth [8]. Dietary supplementation of amaranth hay may improve the oxidation resistance of dairy cows. As an important roughage resource, alfalfa hay is widely used in the ration of dairy cows, particularly for lactating cows. In general, the cost of alfalfa hay in roughage occupies a higher percentage of feeding cost. Alfalfa hay resources are limited, and substantial imports are needed every year in China. Therefore, it is urgent to develop roughage resources which can replace alfalfa in the ration of dairy cows under modern large-scale intensive dairy farming. A previous study used Boer goats with permanent ruminal fistulas as experimental animals and found that the ruminal effective degradability of dry matter (DM) and CP were approximately 67% and 75%, respectively [9]. However, the information of amaranth hay replacing alfalfa hay in the diet of dairy cows remains scarce. Hence, we hypothesized that a reasonable dietary substitution of amaranth hay for alfalfa hay might not affect production performance of lactating cows. The current study was carried out to evaluate the effects of diet containing different levels of amaranth hay instead of alfalfa hay on milking performance, ruminal fermentation, nutrient digestibility, and serum parameters in lactating Holstein cows.

## MATERIALS AND METHODS

### Animal ethics statement

All procedures involving animal care and management used in this experiment were authorized by the Institutional Animal Care and Use Committee of Guangdong Ocean University (Zhanjiang, Guangdong, China).

### Preparation of amaranth and alfalfa hay

The alfalfa hay was imported from the State of California, USA. The whole-plant amaranth at heading stage was harvested with a reaping hook to a 5-cm stubble height and cut into fragments of 4-cm in length by a forage chopper (New Shengtai Machinery Manufacturing Co. Ltd., Jining, China). Next, fresh amaranth forages were naturally dried (approximately 3 d) to make air-dried samples. Chemical composition of alfalfa and amaranth hay are shown in Table 1.

### Experimental animal and diet and feeding management

The animal experiment was performed at a commercial dairy farm that has approximately 8,000 milking cows. Forty-five multiparous (parity = 2) Holstein dairy cows with similar body weight (586±18 kg), milk yield (34.68±1.4 kg) and days in milk (72±5 d) were randomly assigned to 3 groups (15 cows in each group): a control group without amaranth hay (CON) and either 50% (AH1) or 100% (AH2) of the dietary alfalfa hay was replaced by an equal level of amaranth hay.

All the cows were provided with total mixed rations which was formulated based on the NRC [10]. To ensure that the three diets were isoenergetic and isonitrogenous, the proportion of oat hay in the diet was increased with the elevation of amaranth hay while the quantity of corn silage in the diet was decreased. Feed ingredients and nutritional levels of experimental rations are described in Table 2. Cows were fed three times daily at 06:30, 14:30, and 22:30 respectively, allowing 5% to 10% orts and had free access to clean water. All experimental animals were mechanically milked three times each day at 05:30, 13:30, and 21:30 in rotary milking parlor. The feeding trial was conducted with a 10-d adaptive phase followed by 60 d experimental period.

### Sample collection

The feed intake was recorded according to the difference of feed offered and refused and converted into dry matter intake (DMI). DMI of each treatment was used to calculate the average daily DMI per animal. The milk yield was collected daily. On d 60, approximately 50 mL of milk samples were obtained three times all day and blended by volume in the ratio of 4:3:3 (morning, afternoon, and evening) for milk composition analysis. The 4% fat-corrected milk (FCM) yield was calculated from the following equation: 4% FCM = 0.4× milk yield+1.5×milk fat production. Feed efficiency was obtained via dividing 4% FCM by DMI.

On d 60, ruminal fluid samples of all animals were collected through a flexible esophageal tube (Anscitech Animal Husbandry Technology Co. Ltd., Wuhan, China) at 4 h after morning feeding. Immediately, the ruminal fluid pH was measured with a pH meter (Jingcheng Instrument Co. Ltd., Qingdao, China). Next, fluid samples were filtered by 4 layers of cheesecloth and preserved in 10 mL centrifuge tubes and stored at 20°C for analysis of ruminal fermentation parameters.

Fecal samples of all cows were collected from d 58 to 60 according to the procedures described by a previous study [11]. Briefly, fecal samples were collected at 6 h intervals from rectums of cows and the sampling time were as follows: d 58, 20:00, 02:00, 08:00, and 14:00; d 59, 18:00, 00:00, 06:00, and 12:00; and d 60, 16:00, 22:00, 04:00, and 10:00. In the meantime, the fresh feed and orts were sampled daily. The fecal samples were mixed per cow and subsampled. All feed, orts, and fecal samples (the 100 g feces were blended with 10 mL of 10% sulphuric acid) were dried at 65°C in a forced-air oven for 48 h to a constant weight. Subsequently, dried samples were ground to pass through a 1-mm sieve (Aizela Electric Appliance Co. Ltd., Ningbo, China) for measurement of nutrient digestibility.

A total of 10 mL blood were sampled from the caudal vein of each cow using evacuated tubes containing no anticoagulant before morning feeding on d 0 and 60. All blood samples were centrifuged at 3,200 rpm and 4°C for 15 min to collect serum, then preserved at −20°C for further analysis.

### Milk composition and ruminal fermentation analysis

The milk components, including protein, fat, lactose, urea nitrogen (MUN) and solids, were determined through an automated multifunctional milk analyzer (Hengmei Electronic Technology Co. Ltd., Weifang, China). Moreover, somatic cell count (SCC) was analyzed with a fossomatic apparatus (Haiyi Technology Co. Ltd., Beijing, China). The ruminal fluid samples were firstly thawed, then the supernatant was obtained by centrifugation at 15,000 rpm for 12 min at 4°C to measure the contents of volatile fatty acid (VFA), ammonia nitrogen (NH_3_-N) and microbial crude protein (MCP) following previous procedures [11].

### Serum sample and nutrient digestibility analysis

The serum biochemical parameters, including total protein (TP), albumin (ALB), globulin (GLB), alanine transaminase (ALT), aspartate transaminase (AST), alkaline phosphatase (ALP), glucose (GLU), triglyceride (TG), and urea nitrogen (SUN) were determined by an automated biochemical analyzer (BS350; Baden Medical Co. Ltd., Nanjing, China). Besides, the concentrations of glutathione peroxidase (GSH-Px), superoxide dismutase (SOD), catalase (CAT), malondialdehyde (MDA), and total antioxidant capacity (T-AOC) were analyzed utilizing commercially available kits (Nanjing Jiancheng Bioengineering Institute, Nanjing, China) according to the instructions.

The feed and fecal samples were analyzed for DM (method 934.01), organic acid (OM, method 942.05), CP (method 984.13), and ether extract (EE, method 954.02) based on the AOAC procedures [12]. Also, the neutral detergent fiber (NDF) and acid detergent fiber (ADF) contents were measured with an ANKOM fiber analyzer (ANKOM A2000i, USA). The acid-insoluble ash content in feed and feces was determined according to the procedure of Van Keulen and Young [13] and used as an indicator for determination of nutrient digestibility [11].

### Statistical analysis

All data were analyzed with one-way analysis of variance procedure of the SPSS statistical software (version 20.0 for Windows; SPSS, Chicago, IL, USA). Each cow was used as the experimental unit. The model for statistical analysis is as follows: Y = μ+T+e, where Y = dependent variable, μ = general mean, T = treatment effect and e = residual error. Orthogonal polynomial contrasts were completed to detect the linear and quadratic effects according to increase in the dietary amaranth hay levels (three groups) of dairy cows. Tukey test was utilized to determine the differences among three treatments. Results were presented as means and standard error of the mean. Statistical differences were considered significant for p<0.05 and as a tendency for 0.05≤p<0.10.

## RESULTS

### Dry matter intake and milk yield

As shown in Table 3, the DMI of CON and AH1 groups was higher (p<0.005) than that of AH2 group. Similarly, the milk yield of AH2 group was reduced (p<0.05) when compared to AH1 group. In addition, the 4% FCM yield in AH1 group tended to be higher than that in AH2 group. Compared with CON group, the ratio of 4% FCM yield to DMI in AH2 group tended to increase by 3.62%.

### Milk composition

Notably, the milk protein, lactose, total solids, MUN and SCC were similar among three groups (Table 4). However, dietary substitution of alfalfa hay by amaranth hay increased (p<0.05) milk fat.

### Rumen fermentation

**Figure 1:**
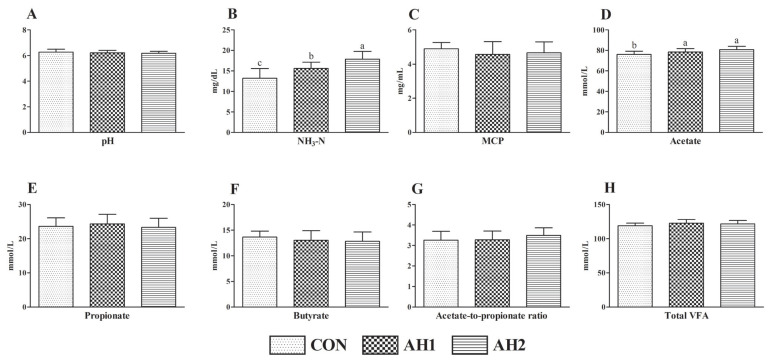
Effects of dietary replacing alfalfa hay by amaranth hay on rumen fermentation of dairy cows. (A) pH; (B) NH_3_-N, ammonia nitrogen; (C) MCP, microbial protein; (D) acetate; (E) propionate; (F) butyrate; (G) acetate-to-propionate ratio; (H) total VFA, total volatile fatty acid. CON, diet with no amaranth hay; AH1, 50% dietary alfalfa hay was replaced by amaranth hay; AH2, 100% dietary alfalfa hay was replaced by amaranth hay. Columns with different small letters mean significant differences (p<0.05).

Ruminal pH was similar and averaged 6.27, 6.22, and 6.18 in CON, AH1, and AH2 groups respectively (Figure 1A). Likewise, there was no significant difference of MCP (Figure 1C), propionate (Figure 1E), butyrate (Figure 1F), total VFA (Figure 1H) concentrations and acetate-to-propionate ratio (Figure 1G) among three groups. Nevertheless, the NH_3_-N (Figure 1B) and acetate (Figure 1D) concentrations of AH1 and AH2 groups were higher (p<0.05) than those of CON group. Moreover, the NH_3_-N concentration in AH2 group was elevated (p<0.05) as compared with AH1 group.

### Nutrient digestibility

**Figure 2:**
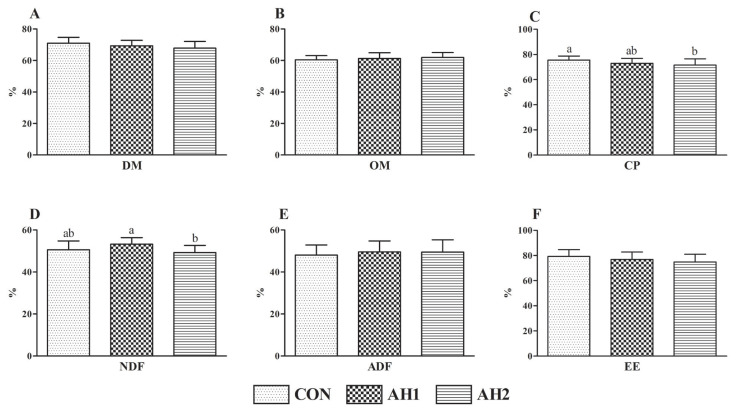
Effects of dietary replacing alfalfa hay by amaranth hay on nutrient digestibility of dairy cows. (A) DM, dry matter; (B) OM, organic matter; (C) CP, crude protein; (D) NDF, neutral detergent fiber; (E) ADF, acid detergent fiber; (F) EE, ether extract. CON, diet with no amaranth hay; AH1, 50% dietary alfalfa hay was replaced by amaranth hay; AH2, 100% dietary alfalfa hay was replaced by amaranth hay. Columns with different small letters mean significant differences (p<0.05).

The OM (Figure 2B), ADF (Figure 2E), and EE (Figure 2F) digestibility did not show significant difference among three groups. However, the CP digestibility (Figure 2C) of AH2 group was lower (p<0.05) than that of CON group. Similarly, the DM digestibility (Figure 2A) of CON group tended to be higher than AH2 group. Compared with AH2 group, the NDF digestibility (Figure 2D) of AH1 group was increased (p<0.05).

### Serum biochemical parameter

On d 0, no obvious difference of all serum biochemical parameters was observed among three treatments (Table 5). Likewise, the concentrations of TP, ALB, GLB, GLU, TG, ALT, AST, and ALP were similar among three treatments on d 60. However, the SUN content of AH2 group was increased (p<0.05) when compared to CON group.

### Serum antioxidant parameter

There was no significant difference of GSH-Px, CAT, SOD, MDA, and T-AOC serum concentrations among CON, AH1, and AH2 groups on d 0 (Table 6). On d 60, the CAT and SOD concentrations were similar among three groups. However, dietary substitution of alfalfa hay by amaranth hay increased (p<0.05) the concentration of GSH-Px in serum of cows. Compared with CON group, the MDA activity of AH1 group was decreased (p<0.05). In addition, the T-AOC activity in amaranth hay groups tended to be higher than CON group.

### Economic benefit

As shown in Table 7, the AH1 group displayed the greatest economic benefit. Compared with CON and AH2 groups, the farming benefit of cow per day in AH1 group was increased by 5.01% and 5.12% respectively.

## DISCUSSION

### Dry matter intake, milk yield, and milk composition

Feed intake is critical for dairy cows to maintain the milking performance. Previous study in fattening lambs found that dietary supplementation with amaranth silage increased the DMI [7]. In the current study, 50% dietary alfalfa hay replacement by amaranth hay had no obvious effect on DMI of dairy cows, whereas 100% replacement significantly reduced the DMI. The chemical composition, especially NDF content, of ration affect DMI of ruminants [1]. Thus, the reduced DMI of AH2 group may be related to high NDF content of amaranth hay. Correspondingly, the cows in AH2 group showed lowest milk yield, illustrating that the 100% substitution ratio might be too much when replacing alfalfa hay with amaranth hay in dairy cows. Different physical characteristics of the experimental feeds such as fiber texture have direct influence on DMI [14]. The increased fiber content in amaranth hay may induce satiety and decrease DMI of dairy cows, then result in reduced milk yield. According to the results of current study, 50% substitution of alfalfa hay by amaranth hay did not affect lactation performance of dairy cows but improved the economic benefit of dairy farming.

Milk composition is the main index to evaluate the quality of milk. A previous study has reported that the substitution of alfalfa hay by different levels of roughage did not affect the milk composition [15]. Our study found that dietary replacement of alfalfa hay with amaranth hay did not affect the milk protein, lactose, total solids, MUN and SCC of dairy cows, which was consistent with previous study [15], suggesting that amaranth hay did not have negative influence on milk composition. However, the amaranth hay treatments significantly increased the milk fat. Milk fat is an important parameter to assess the production performance of dairy cows and can provide energy, fatty acid, and essential nutrients for humans. The milk fat can be synthesized by acetate and butyrate, and high acetate and butyrate contents commonly lead to increased milk fat production [16]. In this experiment, although the butyrate content was not changed among different treatments, the content of ruminal acetate was elevated significantly in amaranth hay substitution treatments, which was matched to the milk fat result.

### Rumen fermentation

In general, the ruminal fermentation characteristics are largely affected by chemical composition of diet. Ruminal pH affects the growth and proliferation of microorganisms and regulates the VFA production within a normal range 6 to 7 [17]. In our research, the ruminal pH of three treatments were within the normal range of 6.18 to 6.27, indicating that amaranth hay had no negative effects on ruminal fermentation of dairy cows. Ruminal NH_3_-N is an intermediate product of dietary protein and non-protein nitrogen degradation and microbial protein synthesis, and its concentration reflects the balance state of protein degradation and synthesis, which is mainly affected by dietary protein degradation, ruminal epithelium absorption, microbial utilization and digesta outflow rate [18]. With the elevation of substitution level, the NH_3_-N concentration was significantly increased. The result could be attributed to the fact that the fiber content of amaranth hay is higher than that of alfalfa hay. Thus, the feed requires multiple rumination to digest, then the retention time of feed in the rumen is increased, resulting in a relatively high NH_3_-N content. In addition, the reduction of dietary fiber content increases the activity of protein-utilizing bacteria, then decreases the NH_3_-N content in the rumen [19]. The fiber structure of amaranth hay and effects of amaranth hay on ruminal microbial community need further investigation.

The acetate in the rumen is mainly produced by microbial fermentation with NDF as substrate [20]. Therefore, in the present experiment, increased ruminal acetate content in amaranth hay treatments could be attributed to the high NDF content. However, although the ruminal acetate concentration was increased in AH1 and AH2 groups, the total VFA concentration was similar among three groups, which may be associated with low nutrient digestibility. Previously, a study has found that the NDF content in the ration had no obvious effect on ruminal total VFA concentration [21], which was in accordance with our study. Moreover, a recent study reported that the acetate-to-propionate ratio had positive correlation with milk fat yield [22]. We did not observe significant difference of ratio of acetate to propionate. However, the ratio of acetate to propionate of three groups was more than 3, thus the milk fat percentage was above 3.9% in all dairy cows. An *in vitro* study demonstrated that the VFA production of amaranth diet was low [23], which was inconsistent with our study. In our experiment, the ruminal fluid samples were collected after morning feeding and might only reflect the ruminal fermentation characteristics at that time. The dynamic patterns of rumen fermentation caused by dietary substitution of alfalfa hay with amaranth hay require an in-depth study.

### Nutrient digestibility

In addition to rumen fermentation, nutrient digestibility directly affects the production performance of cows. A previous study investigated the influence of different roughage on nutrient digestibility of milking cows and found that the nutrient digestibility was reduced after dietary replacement of alfalfa hay with straw (rice or maize) [24], which was basically consistent with our experiment. The nutrient digestibility of dairy cows is commonly affected by feed type, nutritional level, and fiber content [25]. In this study, the 100% substitution ratio treatment reduced the CP and NDF digestibility, which might be related to high NDF content of amaranth hay. These results might explain the lowest milk yield in AH2 group. Furthermore, we found that 50% substitution ratio of amaranth hay displayed a positive effect on NDF digestibility. The positive effect might be associated with a synergistic effect between amaranth and alfalfa, which would then increase the relative abundance of bacteria associated with cellulose degradation, but this mechanism of action needs to be fully elucidated. A recent study found that partial replacement of millet for alfalfa hay did not affect the nutrient digestibility of dairy cows; however, a high ratio of replacement decreased the nutrient digestibility [26], which was in line with our study.

### Serum parameters

The changes of serum biochemical parameters which are closely related to the health of animals can be used to evaluate the physiological metabolism of the body and changes in various organ function [27]. The TP, ALB, GLB, and SUN concentrations in serum reflect protein metabolism, and the serum GLU and TG contents are associated with lipid and energy metabolism. Additionally, the ALP, ALT, and AST concentrations in serum are indicators of hepatic function [27]. In this experiment, the serum concentrations of TP, ALB, GLB, GLU, TG, ALP, ALT, and AST were similar among three groups, indicating that amaranth hay replacement did not have negative effects on health of dairy cows. A previous study found that dietary substitution of corn silage by amaranth silage did not change the serum biochemistry parameters of fattening lambs [7], which was consistent with our research. However, our result showed that 100% substitution treatment significantly increased the serum SUN concentration, suggesting that the nitrogen conversion was low in AH2 cows. The variation of nitrogen conversion was mostly in accordance with CP digestibility, which indicated that the low nitrogen conversion could be partly explained by the decreased CP digestibility in AH2 group. Nousiainen et al [28] reported that increased content of MUN, SUN, and ruminal NH_3_-N suggested a reduced nitrogen efficiency of dairy cows. In dairy cows, an increase of MUN and ruminal NH_3_-N contents and a decrease of nitrogen conversion were observed in cows when replacing cereal straw for alfalfa hay [24]. In the current study, although the MUN was similar among three treatments, considering the high content of ruminal NH_3_-N and SUN in AH2 cows, a decreased nitrogen conversion efficiency should be expected. A previous study in dairy cows also found that high level of substitution of corn silage by amaranth silage increased the SUN content [6]. According to our findings, we speculated that higher NH_3_-N absorption from the rumen and urea production in the liver were mainly responsible for elevation of SUN in AH2 cows. In the present experiment, the DMI, milk yield, feed efficiency and nitrogen conversion were similar between CON and AH1 treatments, which only reduced in AH2 treatment, indicating that the 50% substitution ratio was appropriate when replacing alfalfa hay with amaranth hay in dairy cows.

The serum GSH-Px, CAT, SOD, and T-AOC are the important biomarkers related to antioxidative ability of animals. As a lipid peroxidative product, serum MAD can be used to reflect the oxidative tissue impairment [29]. In our experiment, serum GSH-Px activity was enhanced, and T-AOC activity tended to be increased by amaranth hay treatment, which may be associated with the antioxidant properties of polyphenols and carotene that naturally occur in amaranth [8]. Our finding was in accordance with the previous study which found that laying hens treated with amaranth grain displayed higher antioxidant enzyme activity and lower MDA concentration in serum [30]. The lower serum MDA caused by amaranth hay indicated decreased lipid peroxidation, which had positive effects on metabolism of dairy cows.

## CONCLUSION

The results from the current study showed that replacing alfalfa (50% ratio) hay with amaranth hay in the diet of dairy cows did not have negative effects on DMI, milk yield, milk composition, ruminal fermentation, and nutrient digestibility, but improved milk fat and antioxidant ability of cows. However, 100% substitution ratio showed reduced DMI, milk yield and CP digestibility and increased serum urea nitrogen content. Therefore, according to our findings, the appropriate substitution ratio of alfalfa hay by amaranth hay was 50% to avoid a negative effect on production performance of dairy cows.

## Figures and Tables

**Table 1:** Chemical components of amaranth and alfalfa hay (dry matter basis, %)

Items	Amaranth hay	Alfalfa hay
CP	12.24	20.02
EE	3.02	2.57
NDF	57.71	37.63
ADF	36.07	26.24
Ash	8.87	9.88
Ca	0.86	1.75
P	0.18	0.28

CP, crude protein; EE, ether extract; NDF, neutral detergent fiber; ADF, acid detergent fiber; Ash, crude ash.

**Table 2:** Ingredients and nutrient levels of the experimental diet (dry matter basis)

Items	Treatments^1)^		

	CON	AH1	AH2
Ingredients (%)			
Alfalfa hay	8.80	4.40	0
Oat hay	4.20	5.60	7.00
Amaranth hay	0	4.40	8.80
Corn silage	34.50	33.10	31.70
Corn	16.71	16.71	16.71
Beet pulp	6.28	6.28	6.28
Distillers dried grains with soluble	6.16	6.16	6.16
Soybean meal	11.52	11.52	11.52
Whole cottonseed	4.18	4.18	4.18
Wheat bran	4.15	4.15	4.15
NaHCO_3_	0.43	0.43	0.43
Limestone	1.45	1.45	1.45
Premix^2)^	1.62	1.62	1.62
Total	100	100	100
Nutrient levels (%)			
CP	16.92	16.89	16.88
NDF	32.93	36.21	37.92
ADF	23.21	26.03	28.89
NFC	38.45	39.27	40.11
Ca	0.88	0.91	0.92
P	0.46	0.44	0.43
NE_L_ (MJ/kg)^3)^	6.84	6.83	6.83

CP, crude protein; NDF, neutral detergent fiber; ADF, acid detergent fiber; NFC, non-fibrous carbohydrate; NE_L_, net energy for lactation.

1) CON, diet with no amaranth hay; AH1, 50% dietary alfalfa hay was replaced by amaranth hay; AH2, 100% dietary alfalfa hay was replaced by amaranth hay.

2) The premix provided the following per kg of experimental ration: Vit A 8,000 IU, Vit D 1,200 IU, Vit E 50 IU, Fe 100 mg, Mn 40 mg, Zn 64 mg, Cu 10 mg, I 0.50 mg, Se 0.38 mg, Co 0.16 mg.

3) NE_L_ was a calculated value, while the others were measured values.

**Table 3:** Effects of dietary replacing alfalfa hay by amaranth hay on dry matter intake and milk yield of dairy cows

Items	Treatments^1)^			SEM	p-value		
	
	CON	AH1	AH2		Treatment	Linear	Quadratic
DMI (kg/d)	23.53^a^	23.94^a^	22.49^b^	0.208	0.011	0.033	0.028
Milk yield (kg/d)	35.56^ab^	35.81^a^	34.39^b^	0.253	0.045	0.053	0.106
4% FCM yield (kg/d)	35.17	35.90	34.61	0.246	0.096	0.339	0.051
Feed efficiency	1.49	1.50	1.54	0.008	0.073	0.064	0.381

SEM, standard error of the mean; DMI, dry matter intake; FCM, fat-corrected milk.

1) CON, diet with no amaranth hay; AH1, 50% dietary alfalfa hay was replaced by amaranth hay; AH2, 100% dietary alfalfa hay was replaced by amaranth hay.

a,b In the same row, values with different superscripts differ significantly (p<0.05).

**Table 4:** Effects of dietary replacing alfalfa hay by amaranth hay on milk composition of dairy cows

Items	Treatments^1)^			SEM	p-value		
	
	CON	AH1	AH2		Treatment	Linear	Quadratic
Milk fat (%)	3.93^b^	4.02^a^	4.04^a^	0.013	<0.001	<0.001	0.163
Milk protein (%)	3.41	3.38	3.37	0.012	0.351	0.163	0.724
Lactose (%)	4.96	4.89	5.02	0.052	0.610	0.622	0.391
Total solids (%)	12.93	12.64	12.73	0.146	0.720	0.591	0.546
MUN (%)	14.43	14.84	15.08	0.291	0.688	0.377	0.895
SCC (×10^3^/mL)	18.57	19.65	20.12	0.482	0.413	0.197	0.772

SEM, standard error of the mean; MUN, milk urea nitrogen; SCC, somatic cell count.

1) CON, diet with no amaranth hay; AH1, 50% dietary alfalfa hay was replaced by amaranth hay; AH2, 100% dietary alfalfa hay was replaced by amaranth hay.

a,b In the same row, values with different superscripts differ significantly (p<0.05).

**Table 5:** Effects of dietary replacing alfalfa hay by amaranth hay on serum biochemical parameters of dairy cows

Items	Treatments^1)^			SEM	p-value		
	
	CON	AH1	AH2		Treatment	Linear	Quadratic
Day 0							
TP (g/L)	76.05	77.40	73.49	0.926	0.221	0.261	0.183
ALB (g/L)	29.64	28.61	30.25	0.566	0.498	0.663	0.275
GLB (g/L)	46.41	48.79	43.24	1.131	0.133	0.248	0.098
GLU (mmol/L)	3.57	3.40	3.29	0.072	0.294	0.123	0.832
SUN (mmol/L)	3.21	3.09	3.44	0.075	0.150	0.195	0.142
TG (mmol/L)	0.132	0.163	0.149	0.011	0.509	0.521	0.335
ALT (U/L)	32.66	29.65	30.90	0.695	0.209	0.301	0.150
AST (U/L)	88.48	88.17	85.43	0.989	0.392	0.215	0.567
ALP (U/L)	61.75	63.19	59.17	0.831	0.135	0.202	0.120
Day 60							
TP (g/L)	73.28	76.37	74.65	0.759	0.255	0.461	0.139
ALB (g/L)	30.67	31.78	32.24	0.597	0.554	0.293	0.802
GLB (g/L)	42.61	44.59	42.41	0.752	0.434	0.913	0.200
GLU (mmol/L)	3.41	3.47	3.37	0.060	0.781	0.774	0.523
SUN (mmol/L)	3.14^b^	3.25^ab^	3.43^a^	0.043	0.022	0.006	0.665
TG (mmol/L)	0.125	0.112	0.118	0.005	0.539	0.554	0.349
ALT (U/L)	31.93	31.94	29.61	0.524	0.111	0.069	0.286
AST (U/L)	91.28	89.56	87.73	0.806	0.202	0.075	0.973
ALP (U/L)	55.77	55.86	54.65	0.710	0.749	0.528	0.675

SEM, standard error of the mean; TP, total protein; ALB, albumin; GLB, globulin; GLU, glucose; SUN, serum urea nitrogen; TG, triglyceride; ALT, alanine transaminase; AST, aspartate transaminase; ALP, alkaline phosphatase.

1) CON, diet with no amaranth hay; AH1, 50% dietary alfalfa hay was replaced by amaranth hay; AH2, 100% dietary alfalfa hay was replaced by amaranth hay.

a,b In the same row, values with different superscripts differ significantly (p<0.05).

**Table 6:** Effects of dietary replacing alfalfa hay by amaranth hay on serum antioxidant parameters of dairy cows

Items	Treatments^1)^			SEM	p-value		
	
	CON	AH1	AH2		Treatment	Linear	Quadratic
Day 0							
GSH-Px (U/mL)	108.57	101.50	104.89	1.687	0.234	0.339	0.158
CAT (U/mL)	7.89	6.84	7.63	0.293	0.318	0.716	0.144
SOD (U/mL)	30.90	33.17	30.58	0.666	0.227	0.839	0.089
MDA (nmol/mL)	7.82	7.38	6.92	0.220	0.256	0.101	0.983
T-AOC (U/mL)	5.81	6.04	5.74	0.115	0.553	0.806	0.292
Day 60							
GSH-Px (U/mL)	107.25^b^	115.80^a^	117.40^a^	1.761	0.037	0.017	0.331
CAT (U/mL)	5.72	6.25	6.15	0.112	0.121	0.117	0.177
SOD (U/mL)	39.38	35.94	36.69	0.723	0.124	0.127	0.169
MDA (nmol/mL)	6.68^a^	5.94^b^	6.11^ab^	0.127	0.040	0.059	0.080
T-AOC (U/mL)	6.04	6.67	6.70	0.129	0.057	0.053	0.263

SEM, standard error of the mean; GSH-Px, glutathione peroxidase; CAT, catalase; SOD, superoxide dismutase; MDA, malondialdehyde; T-AOC, total antioxidant capacity.

1) CON, diet with no amaranth hay; AH1, 50% dietary alfalfa hay was replaced by amaranth hay; AH2, 100% dietary alfalfa hay was replaced by amaranth hay.

a,b In the same row, values with different superscripts differ significantly (p<0.05).

**Table 7:** Effects of dietary replacing alfalfa hay by amaranth hay on economic benefit of dairy cows farming

Items	Treatments^1)^		

	CON	AH1	AH2
Feed cost (dollar/per cow)	12.04	11.73	11.35
Milk income (dollar/per cow)	21.23	21.38	20.53
Economic benefit (dollar/per cow)	9.19	9.65	9.18

1) CON, diet with no amaranth hay; AH1, 50% dietary alfalfa hay was replaced by amaranth hay; AH2, 100% dietary alfalfa hay was replaced by amaranth hay.
